# Fabrication of Capsaicin Loaded Nanocrystals: Physical Characterizations and In Vivo Evaluation

**DOI:** 10.3390/pharmaceutics13060841

**Published:** 2021-06-07

**Authors:** Barkat Ali Khan, Furqan Rashid, Muhammad Khalid Khan, Saad Saeed Alqahtani, Muhammad Hadi Sultan, Yosif Almoshari

**Affiliations:** 1 Drug Delivery and Cosmetic Lab (DDCL), Gomal Center of Pharmaceutical Sciences, Faculty of Pharmacy, Gomal University, D.I.Khan 29050, Pakistan; Ffurqanrashid7676@yahoo.com (F.R.); khalid.gomalian@gmail.com (M.K.K.); 2 Department of Clinical Pharmacy, College of Pharmacy, Jazan University, Jazan 45142, Saudi Arabia; ssalqahtani@jazanu.edu.sa; 3 Pharmacy Practice Research Unit, College of Pharmacy, Jazan University, Jazan 45142, Saudi Arabia; 4 Department of Pharmaceutics, College of Pharmacy, Jazan University, Jazan 45142, Saudi Arabia; mhsultan@jazanu.edu.sa (M.H.S.); yalmoshari@jazanu.edu.sa (Y.A.)

**Keywords:** nanocrystals, capsaicin, solubility enhancement, BCS class II drugs, top down technique

## Abstract

Nano-crystallization is a new emerging strategy to promote the saturation solubility, dissolution rate and subsequent bioavailability of Biopharmaceutical Class II drugs. Capsaicin belongs to BCS class-II drugs having low water solubility and dissolution rate. Nano-crystals (NC) of pure Capsaicin was developed and optimized in order to increase its water solubility, dissolution and further to promote its adhesiveness to skin epidermis layer. NC formulations were subjected to stability studies, droplet size, surface charge, poly-dispensability index, drug content, entrapment efficiency, thermal analysis, surface morphology, crystalline studies, solubility profile, in vitro release and ex vivo permeation studies. In vivo anti-inflammatory assay (Carrageenan-induced paw edema) was performed in Sprague Dawley rats. Nanocrystals loaded with capsaicin showed particle size 120 ± 3.0 nm with surface charge of −20.7 ± 3.5 and PDI was 0.48 ± 1.5. Drug content and entrapment efficiency of T3 was 85% and 90 ± 1.9% respectively. Thermal studies predicted that melting peak of capsaicin was present in the formulation suggested that there was no interaction between active moieties and excipients in NC formulation. Surface morphology confirmed the presence of Nano-size crystals having rough crystalline surface. XRD proved that the capsaicin NC are successfully developed by using high speed homogenization. The solubility of capsaicin was found to be 12.0 ± 0.013 μg/mL in water. In vitro study revealed that 89.94 ± 1.9% of drug was released within 24 h. Similarly, drug permeation was 68.32 ± 1.83%, drug retained in skin was 16.13 ± 1.11% while drug retained on skin was 9.12 ± 0.14% after 12 h. The nanocrystals showed higher anti-inflammatory activity as compared to marketed product (Dicloran^®^). The study concluded that improvement in dissolution rate of capsaicin may potentially provide the opportunities in the development of a much cost-effective dosage forms that will produce improved pharmacological effects, but at low dose as compared to the already available products.

## 1. Introduction

Dermal and transdermal application of several formulations aim to deliver drugs into or through the skin that allows efficient and painless delivery of drugs with minimum side effects [1]. However, some molecules may not possess optimal physicochemical properties such as solubility, dissolution rate, log p, melting point, molecular weight and dose, which make challenging the delivery of drug through skin. Nanoparticles based drug delivery systems have been developed to enhance the topical delivery. These systems include liposomes, ethosomes, transferosomes, nanoemulsions, solid lipid nano particles, niosomes, polymer coated nanoparticles and nanocrystals [1,2].

In the recent years, nanocrystals-based drug delivery systems have received notable attention in topical and transdermal delivery as they have the ability to improve the penetration of poor soluble drugs through the skin [3]. Nanocrystals are pure drug particles having mean particle size less than 1000 nm and primarily composed of a crystalline drug that are stabilized by using suitable polymers or surfactants [4]. They exhibit several advantages such as high drug loading, less quantity of surfactant and polymers, low cost of fabrication, high reproducibility and feasibility of scale up as compared to other nanoparticles [4,5].

Nanocrystals can be fabricated by top-down method, bottom up methods or by combining both methods. Due to reduction in particles sizes, nanocrystals enhance the saturation solubility and due to large surface area, they exhibit higher dissolution velocity [6]. These features create a concentration gradient between the topically applied formulations and the membranes of skin that results in improved passive diffusion [6,7]. Drug particles having sizes in nanometer range provide much deeper penetration into the skin as well as enhanced retention in hair follicles [7]. Similarly, an enhanced adhesiveness with cell membranes surfaces are shown by the nanocrystals which can improve the residence time of formulation in the skin [5].

Numerous researchers and scientists have worked on dermal delivery of several drugs and observed its potential in topical or transdermal penetration [6,7,8,9,10,11,12,13,14,15]. However, to the best of our knowledge, in vivo studies and the topical delivery of drug nanocrystals of capsaicin have not been explored yet.

Capsaicin is the main carotenoid present in the genus Capsicum, which not only gives the characteristic color (e.g., red, yellow and orange) to the capsicum species but also has analgesic, antioxidant and anti-inflammatory effect. The capsaicin traps free radicals like oxygen molecules, peroxyl radicals and reactive nitrogen species [16]. Moreover, capsaicin is also a potent agonist of transient receptor potential vanilloid 1 (TRPV1) receptors having the ability to inhibit substance P which transmits the pain message to the brain from joins and skin. The more the substance P is inhibited, the less pain is perceived and vice versa [17,18].

Capsaicin belongs to BCS class-II drugs which have poor water solubility resulting in poor dissolution rate which leads to low concentration of drug reaches to the plasma [18]. As a result sub-therapeutic response is achieved. Thus, it is necessitating to improve the hydrophobicity, dissolution rate and saturation solubility of capsaicin by using novel nano-crystal formation technique.

To overcome the above-mentioned problems and to promote the anti-inflammatory activity of capsaicin, this study was aimed to fabricate and characterize nanocrystals formulation of capsaicin and to evaluate its anti-inflammatory effects in animal model.

## 2. Materials and Methods

### 2.1. Materials

Capsaicin was kindly gifted by Apple Pharmaceutical, Rawat, (Islamabad), Pakistan. Carbopol 934 (Sigma Aldrich, Darmstadt, Germany), Tween 80/20 (Merck, Darmstadt Germany), Ethanol (Sigma Aldrich, Chimie Sarl, France), Distilled water.

### 2.2. Fabrication of Capsaicin Nano-Crystals

The nanocrystals formulations were first optimized using varying concentrations of active moiety and other ingredients via the top-down technique as previously described by Khan et al. with slight modification [19]. Briefly, varying quantities of carbopol-934 (as in Table 1) were slowly dispersed in distilled water and placed at magnetic stirrer for well mixing (Part A). In another step, 0.5 g of tween 80 was added to distilled water and kept in water bath at 35 °C for 30 min (Part B). Both the parts A and B were mixed and placed at magnetic stirrer for at least 1 h at 1000 rpm. After getting mixture of A and B, Capsaicin was added and stirring continued for 30 min further to get a suspension which was subjected to high speed homogenization (HSH) using (ULTRA-TURRAX, D7813, Germany) homogenizer at 15,000 rpm for 15 min (Two cycles). These fabricated formulations were stored in refrigerator at 2–8 °C before drying in freeze drier (Lyophilizer).

### 2.3. Lyophilization of Nano-Crystals

The freeze solution of nanocrystals kept in plates was subjected to freeze drier (BIOBASE, Germany) for 24 h according to the previous protocols of Khan et al. [19]. Freeze drying is a process in which liquid formulation is converted to solid form in the presence of vacuum and low temperature. After freeze drying of nanocrystals, the solidified form of nanocrystals were evaluated for various characterizations like particle size, zeta potential, PDI, DSC, surface morphological properties, XRD, drug content, solubility, in vitro release, FT-IR and in vivo study.

### 2.4. Physicochemical Characterizations of Capsaicin Nano-Crystals

#### 2.4.1. Stability Studies

In order to select the stable capsaicin nanocrystals and to discard the unstable or metastable ones, the drug loaded nanocrystals were subjected to thermodynamic stability testing, which comprised of heating and cooling cycles. The formulations were kept at 40 °C and 4 °C for 24 h at each temperature and observed for any physical changes. Various aspects of instability of formulations were examined visually. The selected stable formulations were subjected to centrifugation (Scilogex, Rock Hill, SC, USA) for 15 min at 5000 rpm. The stable nanocrystals of capsaicin were selected for further studies [19].

#### 2.4.2. Zeta Potential, Particle Size and Polydispersity Index of Nano-Crystals

Nanocrystals particle size, surface charge and polydispersity index were evaluated by Malvern equipment, UK (Nano series ZS 90). The sample i.e., 1 mg of capsaicin nanocrystals were taken and dispersed in 10 mL distilled water by brief shaking, i.e., for 2 min. The calculation was based on Mie equation of light scattering with the help of computer software system. Room temperature and 90 °C fixed angle was adopted during the analysis [20].

#### 2.4.3. Differential Scanning Calorimetric Studies

The thermal properties of the pure capsaicin and capsaicin nanocrystals were determined by differential scanning calorimetry (Netzsh-200 PC, Germany). Capsaicin nanocrystals (1 mg) was taken and placed on aluminum pan. The reference was an empty pan. The glass transition temperature of the capsaicin nanocrystals were measured at a heating scan rate of 10 °C/min from 0 to 300 °C and nitrogen is used as a purge gas with a flow rate of 20 mg/min. In addition, the melting point and enthalpy of pure drug and capsaicin nanocrystals were determined [19].

#### 2.4.4. Scanning Electron Microscopy

Surface morphology of capsaicin nanocrystals were examined by field emission scanning electron microscope (FE-SEMS3400-N Hitachi). The freeze dried sample of capsaicin nanocrystals was dissolved in 10 mL distilled water. Then, 2 microliter from the prepared nano crystal solution was put on a glass slide. The sample was placed in desiccator for drying purposes. After complete drying, the sample was coated with gold to minimize electrostatic charging during the analysis. The sample was examined at various magnifications power to define the shape and surface morphology [20].

#### 2.4.5. XRD Studies

X-ray diffraction was performed for capsaicin nanocrystals, by means of D8-Bruker (Germany) apparatus. Required conditions included the target (CuKα), voltage (36 Kilovolt) and flow of current was (35 mA). A scheme of diverging, getting and non-spreading slits of 1°, 1°, 1°, 0.15°, in that order was used. In order to process the data, Eva software was used (Brucker package of Evaluation, Germany). The method was using scan rate of 4 degree/min 2θ between 5° and 80° with 2θ [21].

#### 2.4.6. Drug Content of the Nano-Crystals

Drug content of capsaicin nanocrystals was determined using method reported by Basit et al. [21]. Briefly, 1 mg of capsaicin nanocrystals were diluted in 9 mL of ethanol followed by centrifugation for 2 min at 5000 rpm in order to leach out the encapsulated drug. The solution was filtered using 0.45 um nylon membrane and subjected to spectrophotometer at the wavelength of 224 nm. The sum of drug concentration present in the supernatant and sediment gives us the actual drug content [22]. Following equation was used in order to calculate the drug content.

Drug content = Supernatant drug + Sediment drug(1)

#### 2.4.7. Solubility Studies

The saturation solubility is an important parameter of nano-crystals which is to be determined in this studies. For this purposes, optimized nanocrystals of capsaicin was considered for solubility studies. About 1 mg of capsaicin and capsaicin loaded nanocrystals were taken and dissolved in various solvents like distill water, ethanol, methanol, acetone, buffer solution pH 7.4 and pH 5.5 Sample solution was stirred at 100 rpm at 37 °C for 1 h. The samples were filtered using Whatmann filter paper 0.2 μm. Each sample was analyzed at 224 nm by using UV-Spectrophotometer. The experiment was performed in triplicates and results were averaged.

#### 2.4.8. In Vitro Release

In vitro release of capsaicin nanocrystals was examined using Franz diffusion cell according to the previous protocols [23]. Donor and recipient compartment was separated with semi-permeable membrane (Tuffryn Membrane) having pore size of about 0.45 µm. Sodium acetate buffer pH 5.5 was used in receiving chamber. While donor chamber was loaded with 1 g of drug nanocrystals dissolved in sodium acetate buffer pH 5.5. The temperature of the equipment was adjusted about 32 ± 1 °C. One ml of aliquot was regularly withdrawn from the receiving chamber at time interval (0.5 h, 1 h, 2 h, 4 h, 8 h, 16 h and 24 h) and replaced with equal amount of fresh blank acetate buffer. The aliquots were filtered via syringe filter and then analyzed with the help of double beam spectrophotometer (UV-1601, SHEMADZU, Tokyo, Japan). The experiment was performed three time and results were presented in average with standard deviation and finally data were used to plot the graph of concentration against time [21].

#### 2.4.9. Kinetic Models

Weibull kinetic models was applied to in vitro release data in order to evaluate the release mechanism of capsaicin from nano-crystal formulation [21].

#### 2.4.10. Drug Retention/Permeation Study

Ex vivo permeation study was conducted in Franz diffusion cell (IPS technologies, Mumbai, India). The rat skin was placed on the surface of receptor compartment which was filled with phosphate buffer pH 7.4 and placed on a magnetic stirrer. The stirring speed was set at 150 rpm. The donor compartment was loaded with 1 g of prepared nanocrystals formulation. The temperature was maintained at 35 ± 2 °C throughout the experiment. A sample of 1 mL was taken from the receptor compartment while replacing the same with phosphate buffer. Samples were taken at specified intervals for 12 h and analyzed with the help of UV spectrophotometer (UV-1601, SHEMADZU, Tokyo, Japan) at 280 nm. At the end of the ex vivo permeation studies, the drug retained on the skin was determined according to the previous study conducted by Karri et al. [22]. Briefly, the skin was washed with methanol and filtered through 0.45 μm filter paper and analyzed for drug content using UV spectrophotometer. Similarly, drug retained in the skin was estimated by cutting the skin into small pieces, soaked in methanol and centrifuged at 5000 rpm for 15 min. The supernatant obtained was filtered and analyzed using UV spectrophotometer. All the studies were performed in triplicates and results were averaged.

#### 2.4.11. Vibrational Analysis

Capsaicin loaded nanocrystals and blank formulation were subjected to ATR-FTIR (Bruker, Germany) analysis. Nano-crystal formulation (1 mg) was placed on the ATR-FTIR diamond crystals and clumped the sample with sample holder tightly at about 30N force. The sample was scanned for recording the spectra. The spectra were recorded at room temperature in 4000–400 cm^−1^ range at a resolution of 4 cm^−1^. The procedure was repeated three times for each sample and results were averaged [22].

### 2.5. In Vivo Studies

#### 2.5.1. Animals

Sprague-Dawley rats (*n* = 12) having weight 170–190 g of either sex were used as test animals. The rats were divided into three groups having 4 rats in each group and maintained at temperature of 25 ± 3 °C with a 12 h/light cycles for two weeks prior to the experiments [23]. The rats were kept under standard laboratory conditions having free access to food and water.

#### 2.5.2. In Vivo Anti-Inflammatory Activity

The in vivo anti-inflammatory activity of capsaicin loaded nanocrystals (in soft paraffin based) was determined according to protocols followed by Madeha et al. with slight modification [24]. For this purpose, edema was induced in the right hind paw, using 0.1 mL 1% *w/v* carrageenan injection in the sub-plantar region of rats. The capsaicin formulation and the standard marketed emulgel of diclofenac sodium (Dicloran^®^ Sami Pharmaceuticals, Karachi, Pakistan) were applied topically on rats 30 min before the administration of carrageenan injection. Using plathysmometer the paw edema volume was measured immediately after carrageenan injection (Zero time) and then at intervals of 30 min, 60 min, 90 min and 120 min, 150 min and 180 min [24].

#### 2.5.3. Treatment Protocols

Group 1 was control group that was treated with 1% carrageenan injection only.

Group 2 was test group that was treated with capsaicin loaded nanocrystals in soft paraffin + Carrageenan injection.

Group 3 was standard group treated with topical marketed emulgel of Diclofenac sodium + Carrageenan injection.

The percent inhibition of paw edema in capsaicin nanocrystals treated test group was compared with control group (Carrageenan treated group) and standard group and following equation was used to calculate % inhibition:

(2) $$\%\;\text{inhibition}\;\mathrm{of}\;\text{drug}=\frac{Vc-Vt\;}{Vt}\times \;100$$

*Vc*—Inflammatory increase in paw edema volume of control group

*Vt*—inflammatory increase in paw edema volume of test group

#### 2.5.4. Statistical Analysis

Research data were analyzed by using various statistical tools like student *t*-test, Pearson correlation and ANOVA (One Way Analysis). All the experiment were performed in triplicates and results were averaged.

## 3. Results and Discussion

### 3.1. Surface Charge, Size and Polydispersity Index of Capsaicin Nano-Crystals

In formulation, droplet size is an imperative parameter used for the distribution of drugs [25]. Nano-crystals size along with polydispersity ratio greatly affects the important characteristics like drug release kinetics, drug distribution, drug permeation through topical route [19]. Size and charge of nano-crystals along with its poly dispersity ratio was evaluated by Malvern apparatus. Particle size, surface charge and PDI of blank nano-crystal formulations T1 and Capsaicin loaded nano-crystals T2 and T3 (Stable formulation) has been shown in Table 2. Formulation developed without active drug (capsaicin) showed droplet size of 130.5 ± 1.7 nm with surface charge −12.7 ± 2.4 nm and PDI is 0.45 ± 0.2. When the capsaicin was added to the formulation (T2), the size of nano-crystals slightly increases 150 ± 2.8 nm with high surface charge −15.2 ± 4.1 and PDI is 0.36 ± 0.4. While in third formulation T3, particle size of nano-crystals was reduced 120 ± 3.0 nm with surface charge of −20.7 ± 3.5 and PDI is 0.48 ± 1.5. Increase in size of nanocrystals was associated with addition of capsaicin in T2 formulation. The reduction in size in T3 formulation of nano crystal is due to increase in the span time of mixing [19]. Smaller particle size due to prolong mixing time is responsible for higher stability of the formulation as indicated in surface charge (−20.7 ± 3.5) of the respective nanocrystals [4]. All the developed formulations show longer stability due to high value of zeta potential as reported by Simunkova et al [26]. The zeta potential of T3 formulation is maximum (−20.7 ± 3.5), which shows greater repulsive force due to same charges between the nano-crystals and thus promote the stability of formulation [19].

### 3.2. Drug Content and Entrapment Efficiency

Drug content of capsaicin loaded nano-crystal formulations is given in Table 3. T2 and T3 formulation prepared with 200 mg of capsaicin. The drug content of T2 formulation was found to be 82.5% while T3 was 85%. The encapsulation efficiency of T_2_ and T_3_ was 85 ± 3.4% and 90 ± 1.9% respectively. The results of drug content and encapsulation efficiency and encapsulation efficiency of the nano-crystal system designed in the study are suitable for pharmacological action [19]. The high drug content in the formulation showed that drug is properly loaded within the formulation and exhibited less degradation and handling error [22]. T3 formulation of nanocrystals is considered as an optimized formulation on the basis of stability, drug content and entrapment efficiency parameter, which is proceeded further for various characterization tests.

### 3.3. Differential Scanning Calorimeter

**Figure 1:**
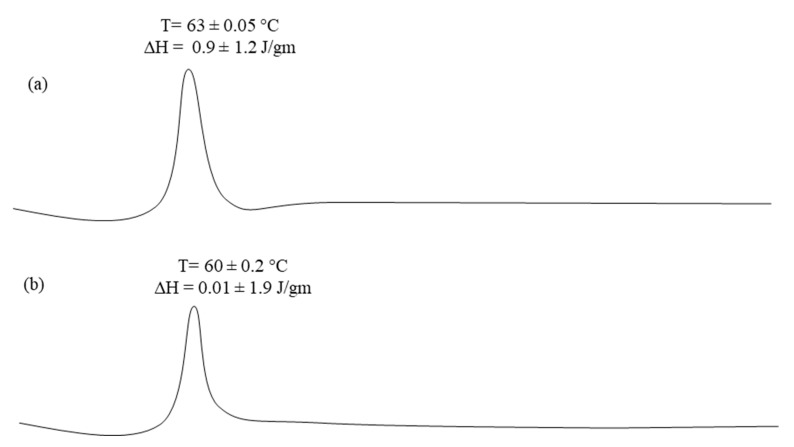
DSC thermo grams of (**a**) Pure Capsaicin and (**b**) T3 Capsaicin loaded nano-crystal formulation.

DSC thermograms of pure capsaicin and capsaicin loaded nano-crystals (T3) are presented in Figure 1a,b respectively. Capsaicin exhibited a sharp endothermic peak at 63 °C ± 0.05 with required enthalpy of 0.9 ± 1.2 (Figure 1a). The nano-crystal loaded with capsaicin showed endothermic peak of capsaicin with low intensity of melting point 60 ± 0.2 °C and its enthalpy is 0.01 ± 1.9 J/gm (Figure 1b). The lowering of melting temperature may be due to the presence of excipients and dilution of drug with various inactive materials [7]. Melting peak of capsaicin was present in the formulation suggested that there was no interaction between active moieties and excipients in nano-crystals formulation [20]. The melting peak of capsaicin in nano-crystal formulations required enthalpy of about 0.01 ± 1.9 J/gm, which clearly indicated that the crystalline nature of the drug was retained in the formulation. This designates possible solid state conversion of the drug [20].

### 3.4. Scanning Electron Microscopy

**Figure 2:**
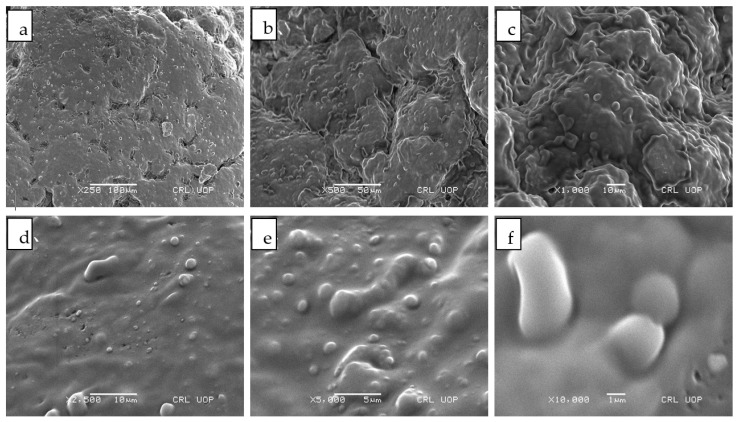
SEM images of T3 Formulation of Capsaicin loaded Nanocrystals (**a**) 250× (**b**) 500× (**c**) 1000× (**d**) 2500× (**e**) 5000× (**f**) 10,000×.

Surface morphology of nano-crystals loaded with capsaicin (T3) were evaluated using high power scanning electron microscope. Figure 2a revealed that the surface morphology of capsaicin nano-crystals at 250 resolution power and Figure 2b–f demonstrates the morphology at magnification power of 500× 1000×, 2000×, 2500×, 5000× and 10,000× respectively. Surface morphology demonstrated that rough crystals surface of capsaicin was appeared in Figure 2a. Solid crystals particles are aggregated and showed crystal of capsaicin within the formulation. Surface morphological properties of nano-crystals confirmed the crystal size which ranges from 130 nm to 120 nm [24]. In Figure 2f at higher magnification power, surface morphology of nano-crystals is more clearly visible. Nano-size crystals were seen in the figure having smooth crystalline surface showing homogenous dispersion of crystal within the formulation [25]. The average size of the capsaicin nanocrystals was found to be less than 200 nm, which was further supported by the results of droplet size analysis.

### 3.5. XRD Studies

**Figure 3:**
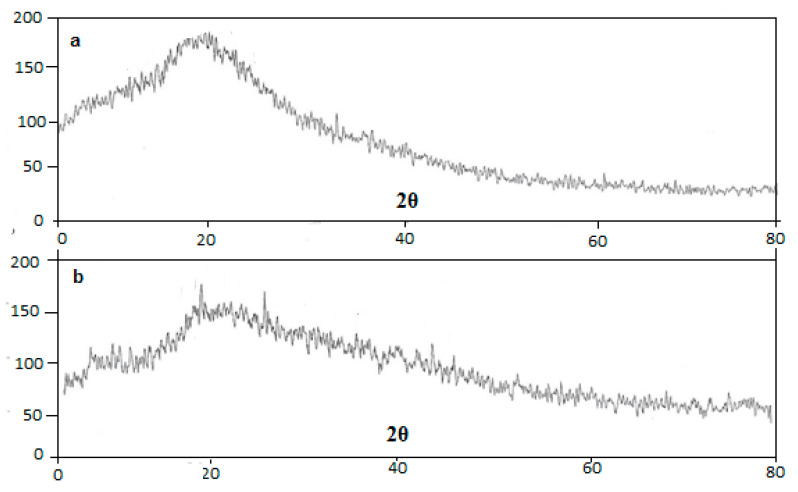
XRD thermo-gram of (**a**) Pure Capsaicin and (**b**) Capsaicin loaded nano-crystal formulation.

XRD graph of the pure capsaicin and capsaicin loaded nanocrystals (T3) is represented in Figure 3a,b, respectively. Pattern of pure capsaicin and capsaicin loaded nanocrystals was studied in the 2θ range between 5° to 80° in scan speed of 4 °C/min. The XRD pattern of pure capsaicin revealed that peak of drug was at 2θ = 15.6857, 17.5783, 23.8135, 25.0052, 26.2619, 27.2455, 29.1017 and 42.6894 indicating presence of crystalline nature. Diffraction patterns of capsaicin and Carbopol were observed at 2θ = 15.8295, 20.8869, 21.3359, 30.9059, 33.6395, 34.9483, 35.4005, 42.9524, 44.8374 and 53.8326 with similar peaks for coarse drug [23]. Patterns observed for capsaicin and nanocrystals loaded with capsaicin were found to be quite similar, which may be due the non-homogenous mixing of the drug to the inactive moieties of formulation like Carbopol. Moreover, the peak at 2θ = 23 with capsaicin was disappeared due to the presence of Carbopol layer which encapsulated the drug effectively within the core of the nano-crystal formulation [27].

### 3.6. Solubility Studies

The solubility studies of pure capsaicin and capsaicin loaded nanocrystals in different solvents have been presented in Table 4. For analyzing the solubility profile of capsaicin as an active drug and capsaicin loaded nanocrystals, distilled water, ethanol, methanol, acetone, buffer 7.4 and 6.8 were used in order to check the maximum solubility of drug in respective solvent. The solubility of capsaicin was found to be 18.4 ± 0.42 μg/mL in methanol, which is almost 3.0 times greater, compared to acetone 6.3 ± 0.15 μg/mL. The lowest solubility was observed in acetone. This is due to fact that surface area of the prepared nanocrystals was significantly increased (*p* < 0.05), resulting increase in the solubility of capsaicin. It has been extensively reported in literature that hydrophilic inactive ingredients like Carbopol can sufficiently increase the solubility of lipophilic compounds [20].

### 3.7. In Vitro Drug Release

**Figure 4:**
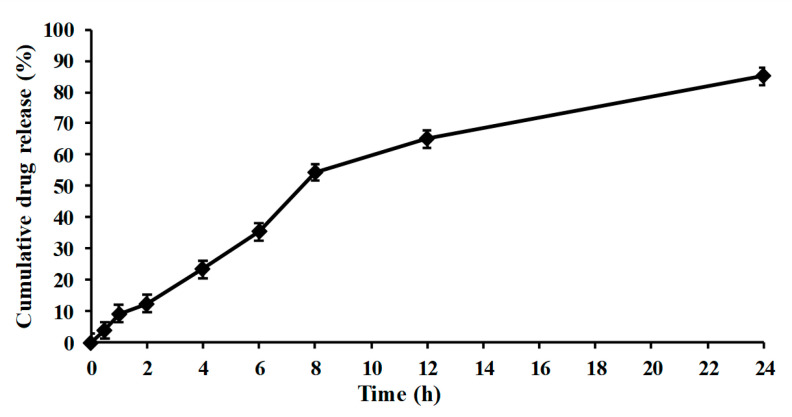
In-vitro release of Capsaicin loaded nanocrystals.

In vitro release pattern of the nano-crystal T-3 formulations is demonstrated in Figure 4. The release pattern of T3 showed delayed release of capsaicin. Cumulative percent results showed that 89.94% drug was released within 24 h of analysis by using Tuffryn membrane [18]. The nano-crystal formulation is responsible for controlled release of capsaicin from the system. Nano-crystal of capsaicin having low aqueous solubility, slows dissolution of drug responsible for less permeation via semi-permeable membrane [28]. Kumar et al in 2019 reported the similar results of drug release. Lower cumulative drug release observed with nanocrystal formulation which could be attributed to crystallization nature. There may be a decrease in concentration gradient and thus in release via permeable membrane due to crystallization [28].

### 3.8. Kinetic Model

**Figure 5:**
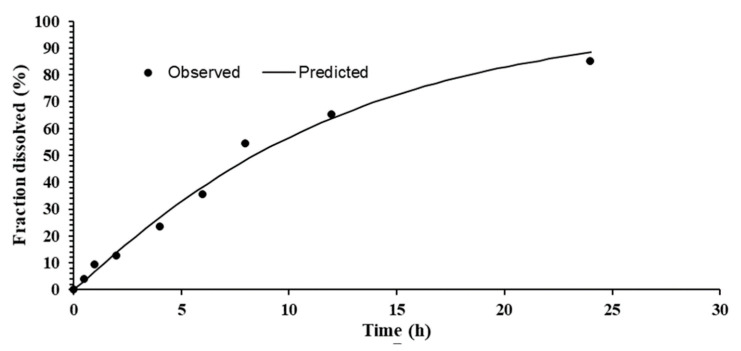
Weibull kinetics model of Capsaicin loaded Nano-crystals.

Drug release kinetics was determined utilizing DD-Solver and by plotting release data in Weibull kinetics model as shown in Figure 5. The results of capsaicin loaded nano-crystals displayed the b > 1 (more than 1), designate that there was slow release of capsaicin from the formulation which is due to the crystalline nature of drug. The crystalline nature of drug is one of the main factor for delay or slow release of active drug from the nano-crystal formulation [20].

### 3.9. Ex Vivo Permeation/Drug Retention Studies

**Figure 6:**
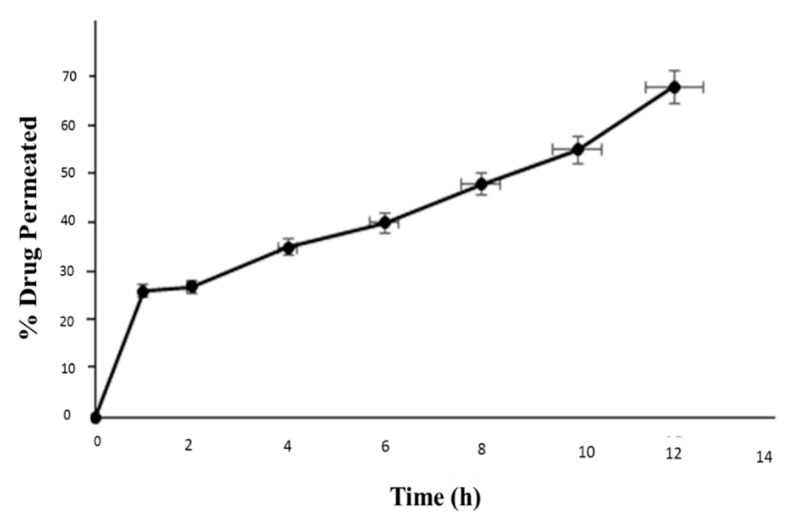
Ex vivo drug permeation of nanocrystal formulation.

The percent drug permeated through from nanocrystal formulation through the skin was found to be 26.21 ± 0.17%, 27.11 ± 0.21%, 35.30 ± 1.10%, 40 ± 1.19%, 48.19 ± 1.48%, 55 ± 1.66% and 68.32 ± 1.83% at time 1 h, 2 h, 4 h, 6 h, 8 h, 10 h and 12 h respectively as shown in Figure 6. The results of % drug permeated (Receptor Compartment), % drug retained on the skin and % drug retained in the skin (Donor Compartment) have been listed in Table 5. The smaller particles size of the nanocrystals enhanced the permeation of capsaicin across the skin thus establishing high concentration gradient on the affected area which will provide efficient drug delivery [22,23]. The pharmacological effects of capsaicin is possible only if larger amount of drug permeates into skin and retained for longer period of time at the affected areas. Thus, the nanocrystals formulations could be promising form of drug delivery for the capsaicin as anti-inflammatory.

### 3.10. ATR-FTIR

**Figure 7:**
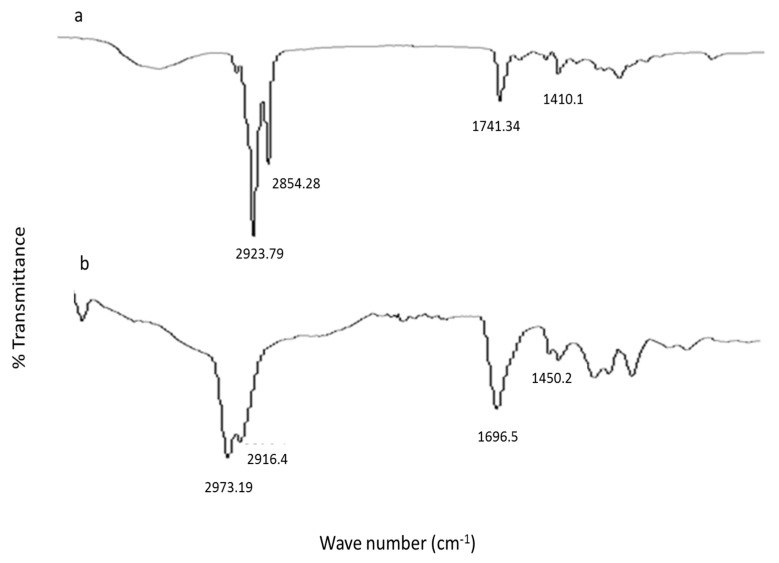
(**a**) FTIR Spectra of unloaded and (**b**) capsaicin loaded nanocrystal formulation.

Nano crystals with and without incorporating capsaicin were subjected to take fingerprint spectrum using ATR-FTIR equipment as shown in Figure 7. Blank nano crystal shows the peak at 2923.79 cm^−1^ and 2854.28 cm^−1^ which are assigned to the C-H group stretching [14]. Carbonyl group stretching was evident at peak 1741.34 cm^−1^. The characteristic peak is at 1410.1 cm^−1^ is due to the C-C stretching as in Figure 7a. The capsaicin nano-crystal exhibited peak at 2973.19 cm^−1^ and 2916.4 cm^−1^. Carbonyl group stretching appear at 1696.5 cm^−1^ and C-C stretching was observed at 1450.2 cm^−1^ Figure 7b [23]. In the formulation spectra of FTIR discloses the absence of characteristic peak of capsaicin. The absence of characteristics drug peak is the main sign of successful drug entrapment inside the core of the nano-crystal formulation [28].

### 3.11. Anti-Inflammatory Activity

In the current study, natural product like capsaicin loaded nanocrystals was evaluated against the inflammation in rats by inducing paw edema using carrageenan. Capsaicin is a natural product obtained from the capsicum and has been rationally employed to treat inflammation, pain osteoarthritis and fibromyalgia [23]. Previously data reported that capsaicin exhibit significant activity by suppressing various inflammatory mediators [24,25]. Thus, on the basis of previous finding the capsaicin is hypothesized that it may exhibit promising synergistic activity if formulated in nanocrystals formulation against the Carrageenan-induced inflammation.

**Figure 8:**
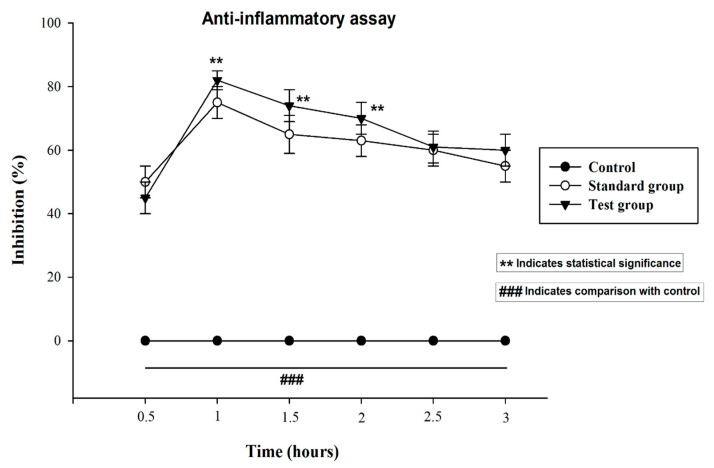
Anti-inflammatory activity of Capsaicin nanocrystals and Dicloran^®^. The control group was left untreated. Standard group was treated with marketed product Dicloran^®^ (Diclofenac sodium). Test group was treated with capsaicin loaded nanocrystals. The data was presented as mean ± SD and analyzed using ANOVA. ^###^ *p* < 0.001 represent control, while ** *p* < 0.02 refers to statistical significance from the control.

The Figure 8 presents the anti-inflammatory activity shown by the capsaicin loaded nanocrystals and diclofenac sodium emulgel. It is observed from results that the group treated with capsaicin nanocrystals exhibited a maximum percent inhibition of edema after 1 h and it was 83 ± 1.80% which is higher than that of marketed emulgel which is 77 ± 1.32%. Thus, edema size induced by the carrageenan injection was significantly inhibited by treatment with capsaicin nanocrystals. Moreover, it was found from statistical analysis using (student t. test) and one way analysis of variance (ANOVA) following Tukey-Kramer tests that capsaicin loaded nanocrystals significantly inhibited and reduced the edema size. The *p* value < 0.05 was considered as significant.

## 4. Conclusions

Capsaicin loaded nano-crystals were successfully formulated in order to promote the aqueous solubility of capsaicin by top down method using high speed homogenization. Capsaicin nanocrystals showed improved solubility at both acidic and basic pH. The improvement in solubility of capsaicin may potentially provide the opportunities in the development of a much cost effective dosage forms that will produce similar or improved pharmacological effects, but at low dose and frequency as compared to the already available products. The anti-inflammatory study in animals have shown enhanced activity as compared marketed product. All the characterizations tests depicted that nano-crystals of capsaicin was successfully designed which acted as a promising vehicle system for the successful carrier of hydrophobic drugs.

## Figures and Tables

**Table 1:** Compositions of Capsaicin Nano-crystals (*w*/*w*).

Formulation Code	Carbopol (*w*/*w*)	Tween-80 (*w*/*w*)	Capsaicin (*w*/*w*)	Water (*w*/*w*)
T1	0.1 g	0.5 g	--	49.4 g
T2	0.5 g	0.5 g	200 mg	48.8 g
T3	1 g	0.5 g	200 mg	48.8 g
T4	1.5 g	0.5 g	250 mg	48.5 g
T5	2 g	0.5 g	250 mg	47.5 g

**Table 2:** Particle size, Surface Charge and Polydispersity index of the Capsaicin incorporated Nano-crystals.

Formulations	Particle Size (nm)	Surface Charge (mV)	PDI
T_1_	130.5 ± 1.7	−12.7 ± 2.4	0.45 ± 0.2
T_2_	150 ± 2.8	−15.2 ± 4.1	0.36 ± 0.4
T_3_	120 ± 3.0	−20.7 ± 3.5	0.48 ± 1.5

**Table 3:** Capsaicin content and encapsulation efficiency.

Formulations Codes	Capsaicin Concentration (mg)	Capsaicin Obtained (mg)	Capsaicin Content %	Percent Encapsulation Efficiency ± SD
T_2_	200 mg	165 ± 1.3 mg	82.5%	85 ± 3.4
T_3_	200 mg	170 ± 1.6 mg	85%	90 ± 1.9

**Table 4:** Solubility of Capsaicin loaded nanocrystals in different solvents.

S.No	Solvents	Solubility of Capsaicin Loaded Nanocrystals μg/mL
1	Water *	12.0 ± 0.013
2	Methanol	18.40 ± 0.42
3	Acetone	6.30 ± 0.015
4	Ethanol	13.84 ± 0.01
5	PBS 7.4	10.0 ± 0.033
6	Acetate buffer 5.5	12.0 ± 0.029

* Water solubility of pure capsaicin was found 0.13 μg/mL.

**Table 5:** % Drug permeated, drug retention in skin and drug retained on skin.

Time Intervals	% Drug Permeated	Retained in Skin (%)	Retained on Skin (%)
0	0	16.13 ± 1.11	9.12 ± 0.14
1	26.21 ± 0.17		
2	27.11 ± 0.21		
4	35.30 ± 1.10		
6	40.00 ± 1.19		
8	48.19 ± 1.48		
10	55.00 ± 1.66		
12	68.32 ± 1.83		

## Data Availability

It can be obtained from the corresponding author on request.

## References

[1] Wu X., Guy R.H. (2009). Applications of nanoparticles in topical drug delivery and in cosmetics. J. Drug Deliv. Sci. Technol..

[2] Gao L., Liu G., Ma J., Wang X., Zhou L., Li X. (2012). Drug nanocrystals: In vivo performances. J. Control. Release.

[3] Sinha B., Müller R.H., Möschwitzer J.P. (2013). Bottom-up approaches for preparing drug nanocrystals: Formulations and factors affecting particle size. Int. J. Pharm..

[4] Shete G., Jain H., Punj D., Prajapat H., Akotiya P., Bansal A.K. (2014). Stabilizers used in nanocrystal based drug delivery systems. J. Excip. Food Chem..

[5] Müller R.H., Zhai X., Romero G.B., Keck C.M., Dragicevic N., Maibach H.I. (2016). Nanocrystals for passive dermal penetration enhancement. Percutaneous Penetration Enhancers Chemical Methods in Penetration Enhancement: Nanocarriers.

[6] Zhai X., Lademann J., Keck C., Müller R.H. (2014). Nanocrystals of medium soluble actives—Novel concept for improved dermal delivery and production strategy. Int. J. Pharm..

[7] Al Shaal L., Shegokar R., Müller R.H. (2011). Production and characterization of antioxidant apigenin nanocrystals as a novel UV skin protective formulation. Int. J. Pharm..

[8] Mitri K., Shegokar R., Gohla S., Anselmi C., Müller R.H. (2011). Lutein nanocrystals as antioxidant formulation for oral and dermal delivery. Int. J. Pharm..

[9] Lai F., Pireddu R., Corrias F., Fadda A.M., Valenti D., Pini E., Sinico C. (2013). Nanosuspension improves tretinoin photostability and delivery to the skin. Int. J. Pharm..

[10] Pireddu R., Sinico C., Ennas G., Marongiu F., Muzzalupo R., Lai F., Fadda A.M. (2015). Novel nanosized formulations of two diclofenac acid polymorphs to improve topical bioavailability. Eur. J. Pharm. Sci..

[11] Vidlářová L., Romero G.B., Hanuš J., Štěpánek F., Müller R.H. (2016). Nanocrystals for dermal penetration enhancement—Effect of concentration and underlying mechanisms using curcumin as model. Eur. J. Pharm. Biopharm..

[12] Hatahet T., Morille M., Hommoss A., Dorandeu C., Müller R., Bégu S. (2016). Dermal quercetin smartCrystals^®^: Formulation development, antioxidant activity and cellular safety. Eur. J. Pharm. Biopharm..

[13] Sinico C., Pireddu R., Pini E., Valenti D., Caddeo C., Fadda A.M., Lai F. (2016). Enhancing topical delivery of resveratrol through a nanosizing approach. Planta Med..

[14] Pyo S.M., Meinke M.C., Keck C.M., Müller R.H. (2016). Rutin—Increased antioxidant activity and skin penetration by nanocrystal technology (smartCrystals). Cosmetics.

[15] Shen C., Shen B., Liu X., Yuan H. (2018). Nanosuspensions based gel as delivery system of nitrofurazone for enhanced dermal bioavailability. J. Drug Deliv. Sci. Technol..

[16] Bode A.M., Dong Z. (2011). The two faces of capsaicin. Cancer Res..

[17] Fattori V., Hohmann M.S.N., Rossaneis A.C., Pinho-Ribeiro F.A., Verri W.A. (2016). Capsaicin: Current understanding of its mechanisms and therapy of pain and other pre-clinical and clinical uses. Molecules.

[18] Gregory S., Devassy R.K. (2016). Integrating TRPV1 receptor function with capsaicin psychophysics. Adv. Pharmacol. Sci..

[19] Khan S., Matas M.D., Zhang J., Anwar J. (2013). Nanocrystal preparation: Low-energy precipitation method revisit-ed. Cryst. Growth Des..

[20] Choi J.-S. (2020). Design of cilostazol nanocrystals for improved solubility. J. Pharm. Innov..

[21] Basit H.M., Cairul M., Mohd I., Ng S., Katas H., Shah S.U., Khan N.R. (2020). Formulation and Evaluation of Microwave-Modified Chitosan-Curcumin Nanoparticles—A Promising Applications Following Burn Wounds. Polymers.

[22] Karri V.V.S.N.R., Raman S., Kuppusamy G., Mulukutla S., Ramaswamy S., Malayandi R. (2014). Terbinafine hydrochloride loaded nanoemulsion based gel for topical application. J. Pharm. Investig..

[23] Ali M., Khan N.R., Hussain Z., Basit H.M., Mahmood S. (2018). Novel Composite pH Controlled Drug Release Hydrogel Containing Dexibuprofen. RADS J. Pharm. Pharm. Sci..

[24] Madeha S., Zahida P., Muhammad R.K. (2017). Evaluation of antioxidant, antiinflammatory, analgesic and antipyreticactivities of the stem bark of Sapindus mukorossi. Complement. Altern. Med..

[25] Simunkova H., Pessenda-Garcia P., Wosik J., Angerer P., Kronberger H., Nauer G.E. (2009). The fundamentals of nano- and submicro-scaled ceramic particles incorporation into electrodeposited nickel layers: Zeta potential measurements. Surf. Coat. Technol..

[26] Verma S., Gokhale R., Burgess D.J. (2009). A comparative study of top-down and bottom-up approaches for the preparation of micro/nanosuspensions. Int. J. Pharm..

[27] Ullah N., Khan S., Ahmed S., Govender T., Faidah H.S., de Matas M., Shahid M., Minhas M.U., Sohail M., Khurram M. (2018). Dexibuprofen nanocrystals with improved therapeu-tic performance: Fabrication, characterization, in silico modeling, and in vivo evaluation. Int. J. Nanomed..

[28] Kumar M., Shanthi N., Mahato A.K., Soni S., Rajnikanth P. (2019). Preparation of luliconazole nanocrystals loaded hydrogel for improvement of dissolution and antifungal activity. Heliyon.

